# Spider (Araneae) Abundance and Diversity Across Land‐Use Gradients

**DOI:** 10.1002/ece3.74261

**Published:** 2026-09-16

**Authors:** Nicola J. Sullivan, Lloyd D. Stringer, Ruth C. Butler, Amanda Black, Nick Waipara, Cor J. Vink

**Affiliations:** ^1^ The New Zealand Institute for Bioeconomy Science Limited Christchurch New Zealand; ^2^ Department of Pest‐Management and Conservation, Faculty of Agriculture and Life Sciences Lincoln University Christchurch New Zealand; ^3^ StatsWork 2022 Limited Lincoln New Zealand; ^4^ Department of Soil & Physical Sciences, Faculty of Agriculture and Life Sciences Lincoln University Christchurch New Zealand; ^5^ The New Zealand Institute for Bioeconomy Science Limited Auckland New Zealand

**Keywords:** biological control, horticulture, integrated pest management, kiwifruit, land connectivity, maize

## Abstract

In agricultural insect pest control, there has been a movement away from broad‐spectrum insecticide use towards more sustainable practises. These include lowering pesticide inputs and reducing the harm on beneficial invertebrates, and keeping a more diverse landscape to allow for a more holistic approach to agriculture. Spiders are the dominant and most abundant invertebrate predators in horticultural growing systems. Despite the known potential of spiders as pest reduction ecosystem service providers, they are generally understudied worldwide. We sampled spider populations in economically important kiwifruit orchards that are adjacent to either low‐disturbance ecosystems (native forest) or high‐disturbance ecosystems (maize), using pitfall traps and active sampling. A total of 2710 spiders were collected from 27 families, representing 64 species and eight functional guilds. The total spider count comprised 2064 (76.2% of total) immature specimens and 646 (23.8% of total) adults. The four most common species (*Tenuiphantes tenuis, Erigone prominens, Leucauge dromedaria, Anoteropsis hilaris*) accounted for more than half of the adults found (341 of 646 adults). Orb web weavers were the most represented functional group, comprising 45.9% of the total catch. Spider abundance and diversity was greatest in the native forest and lowest in the maize crops, with kiwifruit in between. However, spider abundance and diversity were greater in kiwifruit crops that were adjacent to maize crops compared with kiwifruit crops adjacent to native forest. We observed large numbers of immatures along edges of all three land‐use types, whereas densities of adult spiders appeared stable throughout the systems. We conclude there is potential for adjacent land‐use to host a source population of spiders in productive systems. However, more studies are needed to ascertain whether factors such as increased landscape connectivity, reduced synthetic management inputs, or within‐crop non‐crop plantings can enhance retention of spiders in recipient habitats.

## Graphical abstract

We sampled spider populations in economically important kiwifruit orchards that are adjacent to either low‐disturbance ecosystems (native forest) or high‐disturbance ecosystems (maize), using pitfall traps and active sampling. A total of 2710 spiders were collected from 27 families, representing 64 species and eight functional guilds. Spider abundance and diversity were higher in kiwifruit crops that were adjacent to maize crops compared with kiwifruit crops adjacent to native forest, indicating limited support for a spill‐over effect for spiders.

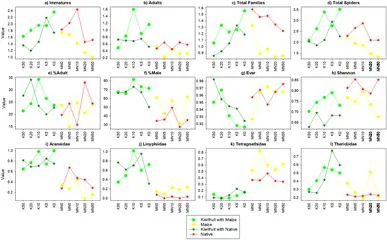

## 1 Introduction

Since the 1940s, using pesticides to control insect pests and intensify agriculture has been the standard practise worldwide (Benamú et al. 2017; Fountain 2022). Although providing economic gains, this practise has also produced many costs: to biodiversity, to the environment, and to human health (Ruberson et al. 1998; Nicolopoulou‐Stamati et al. 2016; Cardoso et al. 2020; Blaise et al. 2022). More recently there has been a push towards more sustainable practises that include lowering pesticide inputs and reducing the harm on beneficial invertebrates, and keeping a more diverse landscape to allow for a more holistic approach to agriculture (Ruberson et al. 1998; Blaise et al. 2022; Fountain 2022; Guaitero and Gutierrez 2024). Many countries have placed restrictions on broad‐spectrum insecticide use, creating the need for alternative pest control options in order to maintain food security and feed the world, particularly at a time of significant climate change and a sixth mass extinction event threatening biodiversity (Barnosky et al. 2011; Cardoso et al. 2020).

The typical intensified agricultural monoculture promotes biodiversity loss (Benamú et al. 2017; Cardoso et al. 2020). Loss of biodiversity simplifies ecosystem services and alters predation and competition interactions, providing space for insect pest plagues to cause damage and monetary losses for growers (Bianchi et al. 2006; Benamú et al. 2017). Retaining or increasing biodiversity can create a more resilient ecosystem that can cope with incoming insect pest disturbance events (Snyder et al. 2006; Senapathi et al. 2015; Måsviken et al. 2023), which are happening more frequently in the age of rapid climate change (Walther et al. 2002; Parmesan 2006). To enable biodiversity retention whilst harnessing the ecosystem services provided by beneficial invertebrates, conservation biological control can be employed to control pest invertebrates, as part of an Integrated Pest Management (IPM) strategy (Barbosa 1998; Ehler 1998; Picchi et al. 2016; Fountain 2022).

Integrated Pest Management is defined as ‘combining knowledge of pests and plants with biological, cultural and chemical measures’ (Hatteland et al. 2023). A significant part of IPM is understanding the role of predators in a growing system to utilise the ecosystem service they provide of reducing pest insect numbers. Spiders are the dominant and most abundant invertebrate predators in horticultural growing systems (Sunderland 1999; Michalko et al. 2019). There are currently over 53,000 species of spiders from 134 families that are known to science (World Spider Catalog 2025). Nyffeler and Birkhofer (2017) found spiders consume between 400 and 800 million metric tons of prey per year worldwide, and > 90% of captured prey is insect or collembola. In a meta‐analysis of 58 published studies, Michalko, Dul'a, et al. (2019); Michalko, Pekar, and Entling (2019) found spiders suppressed agricultural pest insects in 79% of cases, and suppression increased with spider taxonomic diversity.

Collectively, spiders occupy all niches in an ecosystem and can eat all life stages of insect pests (Benamú et al. 2017). Spiders also contribute to pest reduction via non‐consumptive effects (e.g., partial feeding, wounding, trait‐mediated effects) (Nakasuji et al. 1973; Benamú et al. 2017). When studied as individual species, some spiders can be considered to select a certain type of prey when considering their hunting strategy (e.g., ambush hunter, space‐web builder, ground hunter) and niche location within the growing system (e.g., canopy, leaf litter, ground dweller). When a pest is looked at in a growing system, not all spiders may influence its abundance, and indeed the efficiency of any given spider species may depend on factors like the time of year or the life stage of the focus pest (Marc and Canard 1997; Benamú et al. 2017). Such factors highlight the importance of retaining and enhancing a large diversity of spider species to increase the likelihood that one or more of those species will influence populations of the target insect pest(s).

Despite the known potential of spiders as pest reduction ecosystem service providers, they are generally understudied worldwide (Bach et al. 2024), and particularly understudied in Aotearoa New Zealand (Sullivan et al. 2025). When Europe and the United States initiated development of spider‐based biological control programmes, the first step was to improve the knowledge of spider communities in agroecosystems followed by conducting studies to understand the role spiders could play in controlling pests under experimental conditions (Marc et al. 1999). Indeed, understanding how ecosystems function is considered an essential first step before attempting to modify a target system for the desired outcome (Uetz et al. 1999; Fountain 2022).

Edge effects are a common ecological phenomenon studied in biology (Fonseca and Joner 2007; Ries et al. 2017). The term ‘edge effects’ was first coined by Leopold in 1933 (Leopold 1933) and has since been used widely to describe the ecological processes that occur at the boundary between two habitats. In the current study, we are particularly interested in the movement of organisms (namely, spiders) across the boundary of two ecosystems, and the potential access spiders have to complementary resources provided by the ecosystems on either side of the boundary. These are two of the identified mechanisms underlying edge responses identified by Ries et al. (2017).

‘Spillover’ is a term that has increased in usage in ecological settings since the 1980s (Harman and Kim 2024). The word originated from the field of economics and was subsequently adopted in the field of physics, and then ecology. Definitions are not commonly used when reporting spillover effects (Harman and Kim 2024), which is arguably a practice that should be improved. Here we refer to the term ‘spillover’ to mean the movement of organisms (namely, spiders) from a donor habitat into the recipient habitat. This definition of spillover is the most commonly used definition in the agricultural field (Harman and Kim 2024).

In this study we sampled spider communities in kiwifruit orchards (intermediate disturbance ecosystems) in Aotearoa New Zealand that were adjacent to either low disturbance ecosystems (native forest) or high‐disturbance ecosystems (maize crops). We analysed what effect adjacent land‐use has on spider communities within kiwifruit production systems with a view to increasing the ecosystem service spiders provide to pest control in kiwifruit (and extrapolation to other) crops whilst maintaining spider biodiversity for resilience. Specifically, we analysed the effect of season, sampling method, land‐use type and edge effect on spider abundance and biodiversity (number of different species). Our research questions were: (1) how do differences in sampling method affect which spider species and guilds are collected? (2) how does spider community composition differ with season, and with land‐use type? (3) do low‐disturbance ecosystems (native forest) host source populations for spiders dispersing into productive landscapes (kiwifruit crops), demonstrating a spillover effect and landscape connectivity? (4) do edge effects between land‐uses result in different spider community assemblages along edge gradients?

## 2 Materials and Methods

Spider sampling was conducted in Te Kaha, Eastern Bay of Plenty, Aotearoa New Zealand in three different land‐uses: native forest, kiwifruit orchards, and maize crops. Sampling took place during four periods: summer (January) and autumn (April) in each of the years 2023 and 2024. Summer and autumn seasons were chosen for spider sampling, as whilst little is known about spider phenology for most species (Lopes Rodrigues et al. 2023), these seasons are when the highest spider populations have been reported in Aotearoa New Zealand and other countries (Clark et al. 2004; Curtis 2019).

### 2.1 Land‐Use Types

Transects were placed across land‐use boundaries, either from kiwifruit orchards (intermediate disturbance) through to native forest (low disturbance), or from kiwifruit orchards into adjacent maize crops (high‐disturbance). The main crops in Te Kaha, Eastern Bay of Plenty, Aotearoa New Zealand are kiwifruit and maize, and there are several crops which border native forest.

#### 2.1.1 Native Forest

The native forest in this study is part of the Raukūmara ranges which extend 110 km in length and 40 km in width. At Te Kaha, the Raukūmara ranges extend down to 1 km from the coast of the South Pacific Ocean. Kiwifruit crops are often grown right next to the Raukūmara forest park, sharing a boundary with this regenerating coastal podocarp‐broadleaved forest. The range of native and non‐native plants observed in the native forest transects used in this study included: hangehange (*Geniostoma ligustrifolium* var. *Ligustrifolium*) (A. Cunn), kahili ginger ( *Hedychium gardnerianum* ) (Sheppard ex Ker Gawl), kānuka ( *Leptospermum scoparium* ) (J.R.Forst & G.Forst), kawakawa (*Piper excelsum*) (G.Forst), māhoe (*Melicytus ramiflorus*) (J.R. & G. Forster), mamaku ( *Sphaeropteris medullaris* ) ((G.Forst) Bernh), māpou (*Myrsine australis*) (A.Rich), nīkau (*Rhopalostylis sapida*) (H.Wendl. & Drude), pigeonwood (*Hedycarya arborea*) (J.R.Forst & G.Forst), puriri (*Vitex lucens*) (Kirk), ponga/silver fern ( *Cyathea dealbata* ) ((G.Forst) Corda), titoki ( *Alectryon excelsus* ) (Gaertn), marbleleaf/putaputawētā (*Carpodetus serratus*) (J.R.Forst & G.Forst), maidenhair fern ( *Adiantum raddianum* ) (C.Presl), and sedge grass ( *Carex pendula* ) (Huds).

#### 2.1.2 Kiwifruit Orchards

The kiwifruit orchards used in this study had 4.5–5 m row spacing and were grown to a height of 1.5–2 m in a standard flat canopy structure. Each kiwifruit orchard was approximately 5–10 ha in size. Inter‐row growth was minimal, apart from one orchard which had ~20 cm grass growth. The six kiwifruit blocks used were under different managers; however, Aotearoa New Zealand has strict kiwifruit growing regulations, including crop protection standards meaning all insect pest management is strictly regulated and audited, resulting in relatively standardised growing practises. All orchards were export‐grade and were growing the same cultivar of kiwifruit, *Actinidia chinensis* var. *chinensis* ‘Zesy002’.

#### 2.1.3 Maize Crops

Each maize crop was approximately 5–10 ha in size. Maize crops are planted in November/December and are fast growing through to a mature height of 2.5–3 m in March/April. Maize rows are planted at 1 m spacing with 0.3 m between plants. The maize is mechanically harvested using a combine harvester around April each year. The soil is turned over after harvest. From harvest through to planting these maize crops are bare soil with only weeds growing if they are not sprayed with herbicide.

#### 2.1.4 Replication and Resource Constraints

The labour and time‐intensive nature of the assessments carried out in this trial meant that a relatively low number of site‐pairs could be serviced. In addition, given the nature of the trial, there were a limited number of possible sites available. Thus, three pairs only per pair type (kiwifruit‐native; kiwifruit‐maize) were established. Results from this trial are therefore tentative, but are nevertheless informative.

### 2.2 Sampling Methods

**FIGURE 1:**
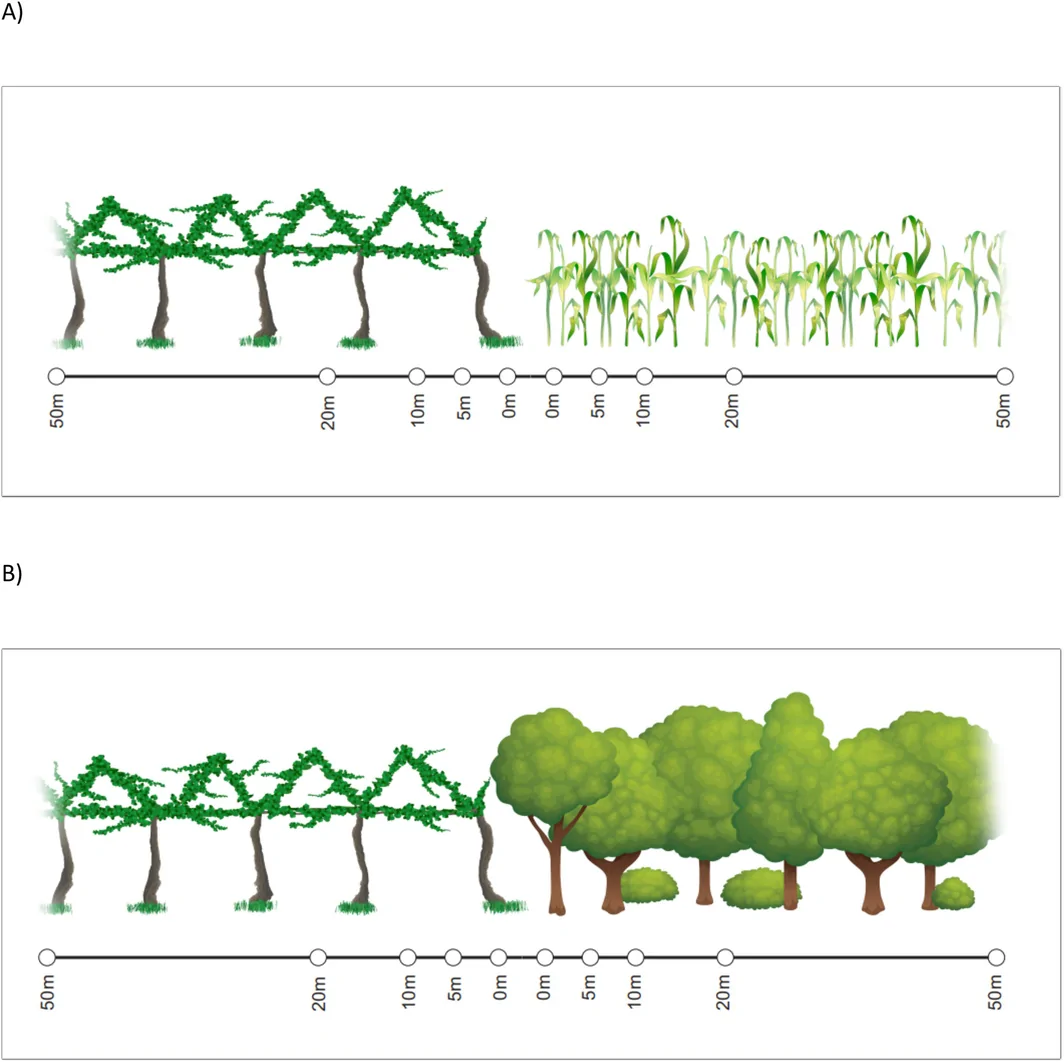
Transect for sampling spiders at each of the three paired sampling sites in Te Kaha, Eastern Bay of Plenty, Aotearoa New Zealand. (A) kiwifruit—maize, (B) kiwifruit—native forest.

At each of the paired sampling sites, a 100 m transect was established: 50 m into the kiwifruit orchard, and 50 m into the adjacent maize crop or native forest (Figure 1). At all the land‐use site boundaries there was a gap separating the two land‐use types. This was sometimes a road (~8 m), a strip of grass, or a shelterbelt of poplar trees (*Populus* spp.). In each case, the interpatch distance was approximately 10 m wide. Where practical, the 0 m was directly opposite the 0 m for the other land use. Native transects were sometimes offset by up to 50 m from their kiwifruit pair to avoid steep hillsides.

At each sampling distance (0, 5, 10, 20, 50 m) five different sampling methods were employed: pitfall trap, active day sampling ground, active day sampling aerial, active night sampling ground, and active night sampling aerial. To avoid interference, three adjacent but spatially separated rows were selected in each kiwifruit orchard. At each distance, the three sampling methods (pitfall, active day, active night [ground and aerial active sampling were inherently vertically spatially separated]) were randomly allocated to the three rows. In the maize and native plots, active day sampling was conducted 3 m to the left of the pitfall traps and active night sampling was conducted 3 m to the right of the pitfall traps.

#### 2.2.1 Pitfall Traps

One pitfall trap (80 mm diameter × 90 mm deep) was dug into the soil so the upper rim of the trap was flush with the ground level at each of the 10 sampling points in all six site‐pairs. In the kiwifruit orchard, pitfall traps were placed in line with the kiwifruit rows to avoid interference with inter‐row machinery or orchard personnel. Pitfall traps were filled with approximately 30 mm of propane‐1,2‐diol (propylene glycol, PG) and covered with a metal tent roof (150 × 200 mm) approximately 20 mm above the ground to prevent debris or rainfall entering the trap. Pitfall traps were left in the ground for 6 days until retrieved and transported back to the laboratory at Lincoln University, Canterbury, New Zealand for spider identification.

#### 2.2.2 Active Timed Collection

Active timed collection was divided into four categories: active day sampling, active night sampling, ground and aerial. Active day sampling was conducted between the hours of 09:00 and 16:00 (at least 2 h before sunset) and active night sampling was conducted between the hours of 21:00 and 02:00 in January and between the hours of 19:00 and 23:00 in April. Night sampling was always started at least 30 min after sunset. During both active day and active night sampling, both a ground sample and an aerial sample were taken at each sampling point. Ground samples were collected from any foliage or soil below the knee, and aerial samples were collected from any foliage or orchard structure above the knee to within arm's reach. At each sampling point, a ~2 m radius was searched by one person for 4 min (resulting in a total of 40 min search time for each transect) and all spiders found were collected into a handheld aspirator or a collection vial. After the 4 min, spiders were euthanised with 70% ethanol and placed into a 21 mL glass jar with label for transportation and future identification.

### 2.3 Spider Identification

All spiders collected were identified morphologically to Family level using Paquin et al. (2010). Adult spiders were further identified morphologically using a dissecting microscope (Nikon SMZ1500) to species level using several references (Guerin 1987; Forster et al. 1990; Vink 2002; Paquin et al. 2010; Vink and Dupérré 2010; Zakharov and Ovtcharenko 2011; Dupérré 2023; World Spider Catalog 2025). Functional guilds were assigned to the spiders collected using Cardoso et al. (2011) (Table S1).

### 2.4 Data Manipulation and Statistical Analyses

All data manipulation and analyses were carried out with Genstat (GenStat Committee 2024a, 2024b).

#### 2.4.1 Richness and Diversity

Smith & Wilson Evenness was calculated for each sample to investigate the spread of spider species across samples and identify the number of rare or common species. Species diversity was calculated using the Shannon–Wiener Index. These were calculated for each sample, where as follows:

*n*_*i*_, *n*_*j*_ is number of individuals for species *i* or *j* (here, families or species).

*S* is number of species present (here, families or species) and is calculated separately for each sample (or combined sample).

*N* is total of individuals: (here, total Spiders).

Smith & Wilson Evenness (Smith and Wilson 1996)

$$\text{Evar}=1-\left(\frac{2}{\pi \;}\right)\arctan\left[\sum_{i}^{s}{\left(\ln n_{i}-\sum_{j}^{s}\ln n_{j}/S\right)}^{2}/S\right]$$

Evar ranges from 0 to 1.

Shannon–Wiener Index (Shannon 1948).

$$H^{\prime }=-\sum_{i}^{S}\frac{n_{i}}{N}\times \ln\frac{n_{i}}{N}$$

This has a lower limit of 0.

For each of these statistics, no calculation can be done if *n*_*i*_, *S* or *N* is 0, since ln(0) and dividing by 0 cannot be carried out.

#### 2.4.2 Analysis of Individual Variables

The five sampling methods consisted of two sampling types (pitfall, active), with four active methods, comprising two heights (ground or aerial) by two sample times (ActiveDayNight: day/night).

There was a total of 400 treatment/fixed effect combinations (5 sampling types × 5 distances × 4 assessments × 2 site pair types; TypePair: maize/kiwifruit, native/kiwifruit) × 2 sites within each pair type (TypeSite: kiwifruit vs. maize or native). At each site, three replicate transects were used, with five distances sampled per transect and each sampling type carried out at each distance. One kiwifruit/native pair was abandoned and a replacement site selected after the first assessment because night sampling could not be carried out. Fourteen sites were therefore used in all.

The fixed effects model formula was thus:

$$\left(\text{TypePair}\times \text{TypeSite}\right)\times \left(\left(\text{Type}/\left(\text{ActiveDayNight}\times \text{ActiveHeight}\right)\right)\times \text{Distance}\right)\times \left(\text{Year}\times \text{Month}\right)$$

In addition to the fixed effects, there were several potentially important random effects. In a site, the five distances × three transects can be considered a ‘plot’ (15 of these per site). For each Active Day/Night plot, there were two sample Heights (ground, aerial). There were four assessments (Year ‘Yr’; Month ‘Mon’) within each sample (Plot).

The full random effects term was therefore:

Pair/Site/Row/Plot/Height/Yr/Mon

These fixed and random models were included in hierarchical generalised linear model analyses (HGLM, Lee et al. 2006). Formal analyses were conducted for the number per sample of immature spiders, adult spiders, total spiders (immature plus adults), and numbers of the four most prevalent families (Linyphiidae, Tetragnathidae, Theridiidae, Araneidae). Also analysed were the numbers of families per sample, the percentage adults of the total found, the percentage male of adults found, and the Smith & Wilson Evenness and Shannon–Wiener index.

Counts (immatures, males, females, adults, total spiders, number of Families plus Families with a total caught of > 300) were analysed with a Poisson distribution for the fixed effects and a gamma distribution for the random effects, both with log‐links. The two percentages (male out of adult spiders; adult out of total spiders) were analysed with a binomial distribution for the fixed effects and a beta distribution for the random effects, with logit links. Smith & Wilson Evenness was analysed with the same model, since it is a proportion (0 to 1). The Shannon–Wiener index was analysed with a normal‐normal HGLM with identity links: this is identical to a restricted maximum likelihood (REML) mixed model analysis.

To assess which random terms were important, an assessment was made using a change in deviance test on dropping a term, as implemented in Genstat's HGRANDOM procedure (GenStat Committee 2024a, 2024b). Two random effects were found to be important: Site and Plots within site. Only these two random effects were retained in the final analyses.

Fixed effects were assessed using a change in deviance test, as implemented in Genstat's HGFIXED procedure (GenStat Committee 2024a, 2024b). A semi‐automatic procedure was used, sequentially adding in groups of terms of increasing complexity (main effects, then 2, 3…8‐way interactions).

Means predicted from the fitted model for each treatment were obtained on the link (log or logit) scale along with associated 95% confidence limits. These were converted back to the original scale (count or percent).

#### 2.4.3 Analyses of Families Together: Canonical Variates Analysis

Due to one kiwifruit/native pair from the first assessment needing to be abandoned and a replacement site selected, in this analysis the data from the first assessment (January 2023) were deleted. Deleting these data removes the main causes of a lack of balance in the data and potential sources of bias. The data were also reduced in size by totalling across the five distances and the three remaining assessments to give 20 treatment combinations (5 trapping methods × 4 pair by crop combinations).

Canonical Variates analysis was used to examine the total spiders for the ten most abundant families (total count ≥ 30; Araneidae, Linyphiidae, Tetragnathidae, Theridiidae, Cycloctenidae, Desidae, Lycosidae, Salticidae, Stiphidiidae, Thomisidae) along with the total over the other less prevalent (‘Minor’) families, where there were fewer than 30 individuals found. Canonical Variates is in essence multivariate analysis of variance (MANOVA) for a simple design. The log (counts+0.5) was examined (to stabilise the variances), with the four land‐use types (kiwifruit next to maize, kiwifruit next to native, maize and native) by the five trapping methods as the ‘groups’. The results are presented as a biplot (Gabriel 1981; Gower and Hand 1996).

## 3 Results

A total of 2710 spiders were collected from 27 families, representing 64 species (Table S2). These were organised into eight different functional guilds according to Cardoso et al. (2011) (Table S3). The total spider count comprised 2064 immature specimens (76.2% of total) and 646 (23.8% of total) adults including 262 females (40.6% of adults) and 384 (59.4% of adults) males. The four most common species ( *Tenuiphantes tenuis* (Blackwall, 1852), *Erigone prominens* (Bösenberg & Strand, 1906), *Leucauge dromedaria* (Thorell, 1881), *Anoteropsis hilaris* (L. Koch, 1877)) accounted for 53% of the adults found (341 of 646 adults). The four most abundant families collected were Linyphiidae, Tetragnathidae, Theridiidae and Araneidae (Table S4). Orb web weavers were the most represented functional group, comprising 45.9% of the total catch (Table S5).

For the main non‐multivariate analyses, evenness was high (mean = 0.94, Table 1), indicating many species were found in multiple sites studied. The Shannon–Wiener index, however, was low with a mean of 0.34 indicating a small number of species dominated the catch, which we can see in the high numbers of the top four species collected (Table S2).

**TABLE 1:** Summary of spider family diversity and richness counts for 1250 combinations of assessment, sampling method, distance and site combinations.

Variable	Mean	Median	Max	Number of missing values^a^
Evar, Smith & Wilson	0.94	1.0	1.0	389
Shannon–Wiener H	0.34	0.0	1.6	389

^a^ Most of the missing values were because of zero counts for individuals, species, or families.

### 3.1 Seasons

**FIGURE 2:**
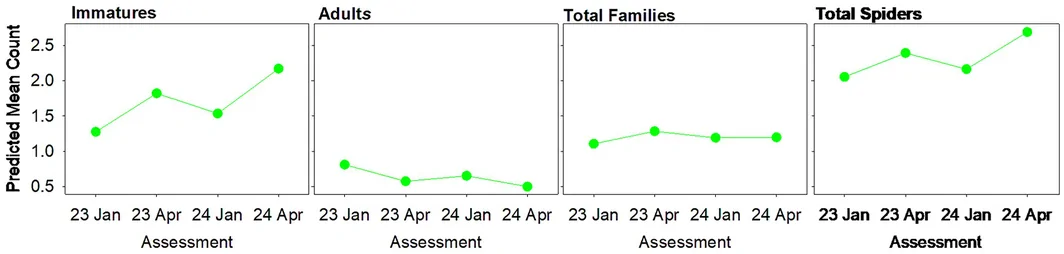
Immature, adult and total spiders, as predicted from the fitted model, caught over time using four different sampling methods in Aotearoa New Zealand kiwifruit orchards, and adjacent maize crops and native forest.

Immature spiders were more abundant in April (Southern Hemisphere autumn) than January (Southern Hemisphere summer) in both years. Conversely, higher numbers of adults were observed in January than April in both years. The total number of spiders showed a similar trend, driven by the immature spider numbers, with higher abundance in April in both years (Figure 2).

### 3.2 Sampling Methods

**FIGURE 3:**
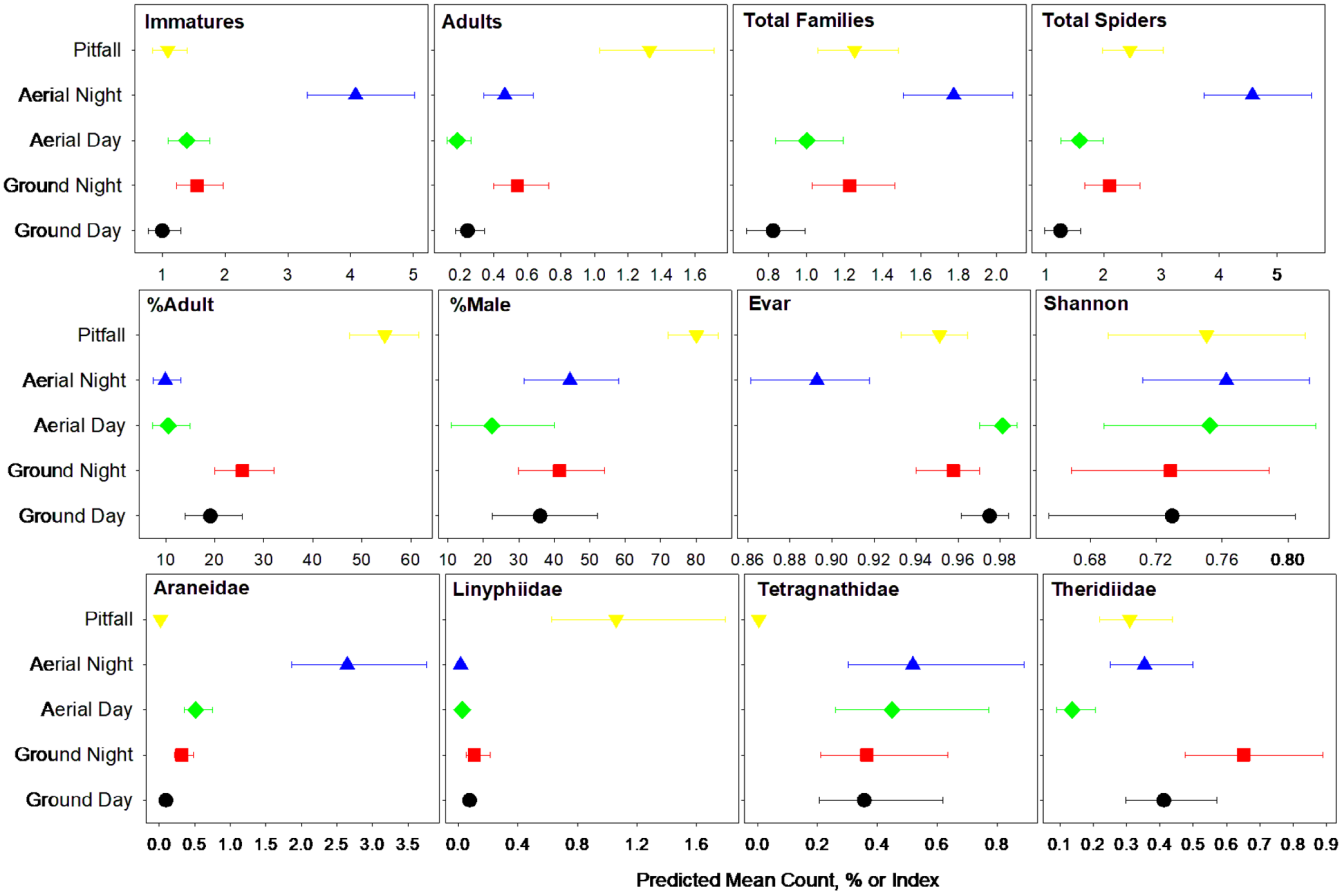
Mean Total numbers of species within the indicated category of spiders caught for the entire trial, percentage that were adults or males, and two diversity statistics (Shannon–Wiener Index [Shannon]; Smith & Wilson Evenness [Evar]) for Aotearoa New Zealand kiwifruit orchards, as obtained from the fitted model, for maize and native forest, by sampling method. Error bars are 95% confidence intervals. The upper limit for a mean of 0 is difficult to obtain so is not shown.

Aerial night sampling resulted in the highest number of immatures (Figure 3), particularly in kiwifruit (Figure S1). Pitfall sampling caught the highest number of adults (Figure 3), also mainly in kiwifruit (Figure S1). Aerial night sampling caught the highest number of total families (Figure 3) and the highest number of total spiders (Figure 3) in all land‐use sites except maize crops (Figure S1, many maize samples did not have an aerial component to sample in April after harvest).

### 3.3 Land‐Use Types

**FIGURE 4:**
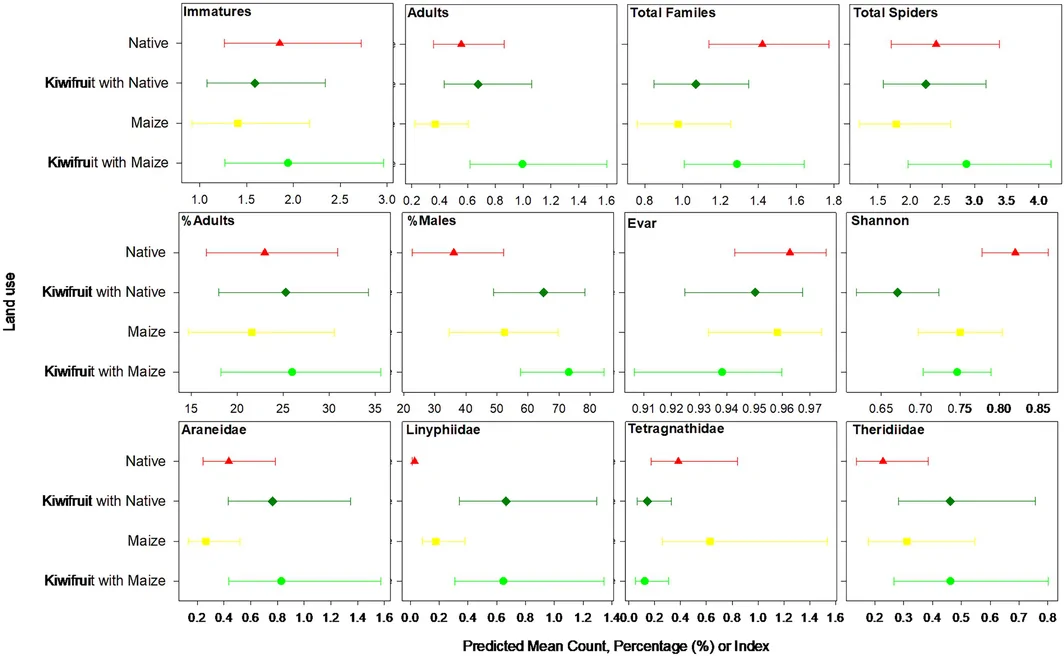
Counts, percentage adult, percentage male, and the two diversity statistics (Shannon–Wiener Index [Shannon]; Smith & Wilson Evenness [Evar]) obtained from the fitted model, for spiders caught over 2 years differentiated by land‐use type. Error bars are 95% confidence intervals.

Spider abundance (total spiders) and diversity (total number of families) were greatest in the native forest and lowest in the maize crops, with kiwifruit in between (Figure 4). Spider abundance and diversity were both higher in kiwifruit crops adjacent to maize crops compared with kiwifruit crops adjacent to native forest (Figure 4).

The Shannon–Wiener index was highest in native forest and lowest in kiwifruit adjacent to native forest (Figure 4).

**FIGURE 5:**
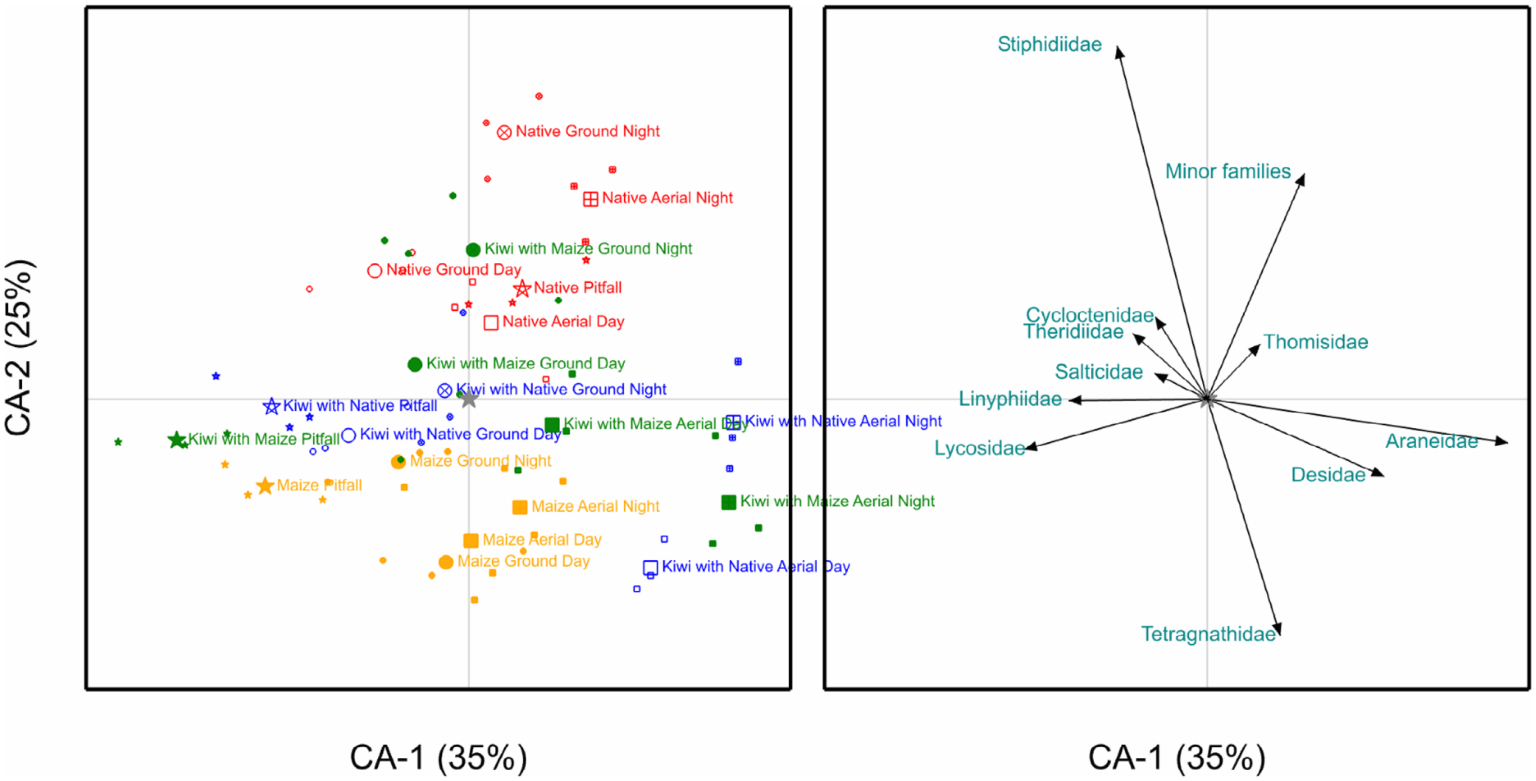
A canonical variates analysis biplot of the total count of spider families caught over 2 years in different land‐use plots. Yellow = maize, green = kiwifruit adjacent to maize, blue = kiwifruit adjacent to native forest, red = native forest. For clarity, data values (small symbols) and means (large symbols) are shown separately (left) from the family dimension vectors (right). The vectors have been multiplied by 4, but the lengths indicate their relative importance. Axes scales are the same for both figures. Minor families = spider families with fewer than 30 individuals caught.

The canonical variates analysis indicated that maize crops and native forests host different families from each other, whereas kiwifruit crops host families from a mix of in between maize and native forest (Figure 5).

### 3.4 Disturbance/Edge Effects

**FIGURE 6:**
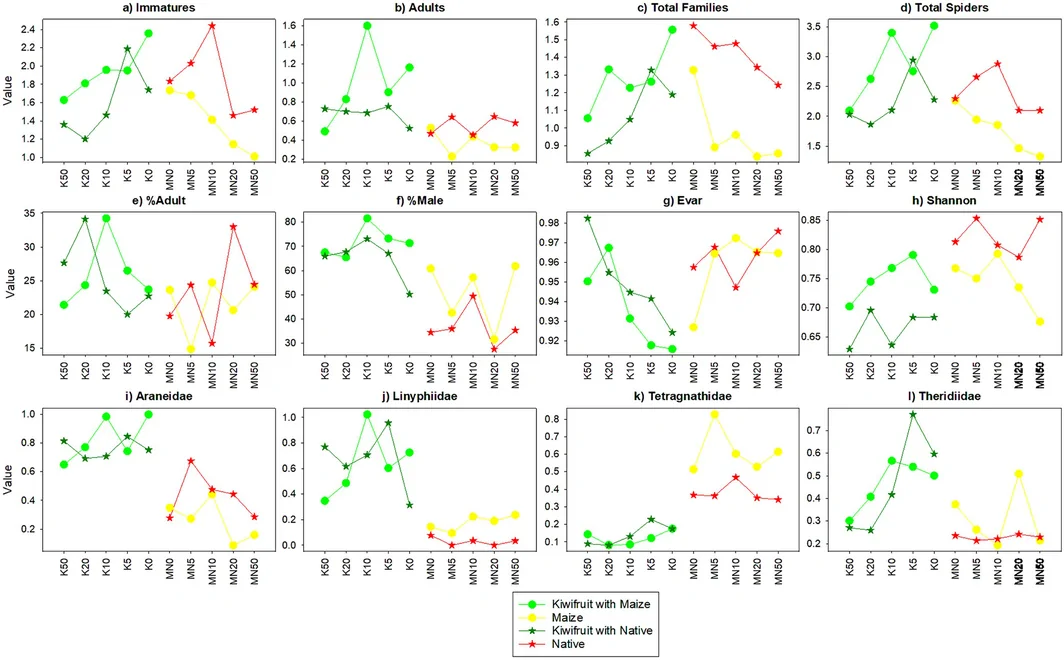
Summary figures of spiders caught in Aotearoa New Zealand kiwifruit orchards and adjacent land‐uses (maize crops or native forest) showing edge effects. K, kiwifruit orchard; MN, maize or native; Shannon, Shannon–Wiener Index; Evar, Smith & Wilson Evenness. Numbers (0–50) = meters from crop edge.

More immature spiders were found at the edges of each land‐use (0 m), with numbers tending to decrease towards the centre of the plots (50 m) (Figure 6a). The number of adults, however, remained relatively stable throughout the transects (0–50 m) for the native, maize, and kiwifruit—native plots (Figure 6b). This trend was less pronounced for kiwifruit—maize plots. Total Families and total spiders also showed an edge effect for all land‐uses, with highest numbers at the edge (0 m) and lowest numbers towards the centre of the plots (50 m) (Figure 6c,d). Species evenness showed the opposite pattern, with lower evenness at the edge climbing to the highest evenness scores at the centre (Figure 6g). The Shannon–Wiener index showed similar diversity between maize and the adjacent kiwifruit crops, but differences between native (high diversity) and the adjacent kiwifruit crops (low diversity) (Figure 6h). The percentage of male adult spiders was higher in both the kiwifruit—maize and kiwifruit—native plots compared with the adjacent maize and native plots (Figure 6f). The percentage of adult spiders (*c.f*. immature spiders) was variable throughout the sampling points in all land‐use types (Figure 6e). When observing the four most common families, Araneidae, Linyphiidae and Theridiidae were all found in higher numbers in both the kiwifruit—maize and kiwifruit—native plots compared with the maize and native plots (Figure 6i,j,l). In contrast, Tetragnathidae was found in much higher numbers in the maize and native plots compared with the kiwifruit plots (Figure 6k).

## 4 Discussion

Many of Aotearoa New Zealand's spider fauna are yet to be described. As of 2010 there were 1126 described spider species in Aotearoa New Zealand, a further 536 known undescribed species and an estimated 330 species undiscovered (Paquin et al. 2010). In the current study, several undescribed species and at least three new to science species were found.

Adjacent land‐use has been found to affect spider communities in productive systems (Bailey et al. 2010; Veres et al. 2013; Beaumelle et al. 2023; de Albuquerque Pereira et al. 2025; Srinivasa Rao et al. 2025), with increased proportions of semi‐natural habitat nearby (Bianchi et al. 2006; Fountain 2022), or within the crop (Blaise et al. 2021; Cargnus et al. 2024) having a positive effect on spider diversity and abundance. Higher levels of natural pest control are known to occur in complex landscapes (Bianchi et al. 2006; Reiff et al. 2024). With et al. (2002) and Bianchi et al. (2006) found landscape patterns specifically affect pest control by natural enemies in productive systems, and do not only depend on within‐crop conditions. Smaller crop patch sizes with high proportions of surrounding non‐crop habitats were found to support higher natural enemy populations and lower pest pressure compared with larger crop patch sizes with little non‐crop habitat (With et al. 2002; Bianchi et al. 2006). Whilst the current study found higher spider abundance and diversity in native forests compared with kiwifruit orchards and maize crops, kiwifruit orchards adjacent to maize crops showed higher abundance and diversity than kiwifruit orchards adjacent to native forests (Figure 6). Indeed, the Shannon–Wiener index (Shannon 1948), indicating species diversity, was highest in native forest and lowest in kiwifruit adjacent to native forest. These results do not support a spillover effect of spiders from adjacent land‐use into kiwifruit orchards. However, the species evenness score of 0.94 could indicate a two‐way spillover effect between the land‐uses, particularly of the most abundant species observed. The high immature numbers at the edges could also indicate a spillover effect of immatures from the adjacent land use, albeit not penetrating more than a few meters.

The current study did not assess the direction of movement of the spiders. It simply observed what spiders were present at set points at four distinct moments in time. We have termed this ‘spillover’ (meaning the movement of organisms (namely, spiders) from a donor habitat (native forest or maize) into the recipient habitat (kiwifruit)) in line with the majority of agricultural studies observing edge effects and spillover (Harman and Kim 2024). This approach gives us insight into what might be occurring in the landscape and allows a foundation for further detailed investigations into the movement processes that may be occurring. Harman and Kim (2024) identify three additional directions of movement often referred to incorrectly as a spillover effect: dispersal, foray loops, and continuous edge movement. Dispersal is when an organism is transferring from a donor habitat, through the focal habitat to a final third habitat. Foray loops are when an organism moves from a donor habitat into a recipient habitat for a resource but then eventually returns to the donor habitat. Continuous edge movement is when an organism resides in two adjacent habitats and moves continuously between them. From our results, it is unlikely that dispersal is important in this system, as numbers of total spiders reduce from the edges (0 m) into the centre (50 m, Figure 6). It is possible that spiders are moving in foray loops – spiders may move from the native forest to the kiwifruit for ample prey supply but move back to the native forest for shelter resource (or vice versa). This could also be a pattern of continuous edge movement – spiders may move between the two habitats continuously to take advantage of the different resources provided. This would follow the Edge Resource Model (Ries and Sisk 2004) whereby organisms occupying adjacent habitats that each have different, complementary resources have the highest densities at the habitat boundary edge. When examining the data between kiwifruit and maize crops (Figure 6), there may be spillover of spiders occurring from the kiwifruit crop as a donor habitat into the maize crop as a recipient habitat.

Higher spider abundance and diversity was observed in the kiwifruit crops adjacent to maize compared with the kiwifruit crops adjacent to native forest. Further to, or instead of spider spillover, a possible explanation could be prey availability. Pest populations can increase with management intensification (Diehl et al. 2013; Birkhofer et al. 2008). Prey populations may therefore be high at maize sites (high management intensity), and prey may be forced to leave the maize crop when the crop is disturbed (e.g., when tilled) (Diehl et al. 2013). Disturbed and migrating prey may arrive at the adjacent kiwifruit crops, providing additional prey items for spiders to consume, therefore resulting in a higher abundance of spiders. Disturbances resulting in prey migration are arguably much less frequent in native forest areas, which could explain the relative difference in spider abundance and diversity between kiwifruit adjacent to maize compared with kiwifruit adjacent to native forest.

Other studies have shown no significant effect of adjacent land‐use on pest management in productive systems (Östman 2002; Ricci et al. 2009; Veres et al. 2013). In a review of how landscape composition affects pest and natural enemy abundance, Veres et al. (2013) found 65% of studies reported significant landscape effects on Conservation Biological Control; while 35% of studies showed no significant effect. The authors highlighted that most of the studies found for the review were from Western Europe and Northern America, which have different climatic conditions (continental V maritime), food production regulations, and flora and fauna to Aotearoa New Zealand (Veres et al. 2013).

Several studies indicated the suitability of productive land itself is more important for the establishment of spider populations than adjacent land‐use (Duffey 1997, 2012; Marc et al. 1999; Haddad et al. 2005; Hogg and Daane 2018). This may explain the findings of the current study, as many disturbances happen in agroecosystems including in kiwifruit orchards (e.g., pruning, harvesting, mowing, agrochemical applications) that have been found to negatively affect spider populations (Marc et al. 1999; Bianchi et al. 2006; Benamú et al. 2017; Reiff et al. 2024). Indeed, others found pesticide pressure in productive systems likely outweighed any beneficial effects provided by landscape context (Ricci et al. 2009; Geiger et al. 2010). In Aotearoa New Zealand, Todd et al. (2016) found greater diversity of natural enemies (including spiders) in organic kiwifruit orchards relative to those managed conventionally but under the principles of IPM, an effect they argued was strongly correlated with less toxic agrichemical sprays. Both Nardi et al. (2019) and Gallé et al. (2022) found forests and woody hedgerows in Italian agricultural landscapes harboured spider species that were not found in the adjacent land uses and that spillover of spider species from the forests into the adjacent agricultural crops was limited, congruent with this current study.

It is worth noting that all the above‐mentioned spider studies (Todd et al. 2016; Nardi et al. 2019; Gallé et al. 2022) studied only ground‐dwelling spiders. All three studies used pitfall traps, with Gallé et al. (2022) being the only study which employed an additional spider sampling method of D‐vac suction sampling, albeit still limited to ground sampling. Although pitfall trapping is the most common form of spider sampling, it can be observed from the current study results that the sampling method used has a large impact on the species and functional guild of spiders caught (Figure 3). Landscape connectivity is defined as “the degree to which the landscape facilitates or impedes movement among resource patches” (Taylor et al. 1993), and an important factor within this is the life‐history of the target organism. The degree to which a species of spider can move between connected landscapes partially depends on its ability to disperse. Ground‐dwelling spider species may have a higher or lower ability to disperse than foliage‐dwelling spider species which are more highly represented in active aerial sampling methods compared with pitfall sampling (e.g., Tetragnathidae and Araneidae, Figure 3). Therefore, to tease out theories of landscape connectivity on spiders, more diverse spider sampling methods should be employed.

Interpatch distance has been found to be the most significant factor in landscape connectivity (With and King 1999; Goodwin and Fahrig 2002). The interpatch distance in the current study was approximately 10 m wide—the width of a road plus operational areas. This interpatch distance, which is typical of Aotearoa New Zealand agricultural settings, is likely an important determinant of the spider species migrating between different landscapes. Indeed, results from the current study showed the highest number of immature spiders at the edge of the kiwifruit orchards compared with the centre (Figure 6a). Ballooning (aeronautic behaviour on silken threads) spiders are known to be able to travel several kilometres (Schmidt and Tscharntke 2005) and are therefore more mobile than many other spider life‐stages or species that do not balloon, and are more frequently found in ecosystems following a disturbance event compared with lower dispersal species (Clark et al. 2004). Ballooning spider populations comprise predominantly immature life stages (Montes and Gleiser 2024). This could indicate that the adjacent land‐use is acting as a source of spiders, of which some species and life stages will be able to disperse from those sources, but then the conditions of the land they arrive in determine whether they make it through to adults and remain functional in that land‐use. Adult numbers were relatively stable throughout the 50 m transect into the kiwifruit orchards in contrast to the number of immature spiders (Figure 6b). Species evenness was also lowest at the edge and highest towards the centre (Figure 6g).

Tetragnathidae was the third most dominant family in this study, with 415 individuals caught over the four sampling periods (predominantly the species *Leucauge dromedaria* ). In contrast, Sullivan et al. (2023) found no Tetragnathidae occupying kiwifruit orchards in Kerikeri, Aotearoa New Zealand, geographically separated from Te Kaha by approximately 600 km of land. Although a large percentage of Tetragnathidae were found in the native forest (low disturbance) and maize crops (high disturbance) of the current study, relatively few were observed in the kiwifruit orchards (Figure 6). Tetragnathidae are highly mobile as immatures, ballooning frequently (Lesar and Unzicker 1978; Blandenier and Fürst 1998). The lack of establishment in adjacent kiwifruit orchards, combined with the lack of Tetragnathidae observed in kiwifruit orchards in another part of Aotearoa New Zealand, shows support for the idea that the conditions of the environment into which a spider species has the opportunity to immigrate to play a more significant role in whether the spider species will establish, compared with the source of that species nearby to be able to immigrate.

Han et al. (2025) demonstrated that floral strips within a pear orchard were more effective than adjacent land‐use at enhancing biocontrol services of arthropod predators. The authors used the tracking markers Rubidium and Strontium to determine movement of arthropod predators from flower strips into pear orchards and found they moved up to 18 m into the crop but were most effective at a distance of 2 m into the crop. Spiders also migrated back into the flower strips after pest psyllid numbers in pear trees had reduced, flower strips acting as a supplementary food supply resource for spiders. Rocher et al. (2024) similarly found inter‐row floral strips in Mediterranean vineyards to be beneficial for increasing pest caterpillar predation by spiders (as well as ladybirds and hoverflies). The current study indicates that Aotearoa New Zealand kiwifruit orchards may not be able to support higher numbers of beneficial spider predators under current growing regimes. However, vegetation strips within kiwifruit orchards may yield better results.

This is the first Aotearoa New Zealand study to analyse the effects of adjacent land‐use on spider communities in productive horticultural systems. We answered four research questions with the following results: (1) spider species and guilds collected were affected by sampling method, with active aerial sampling catching different species and functional guilds from pitfall sampling; (2) spider community composition differed with season and with land‐use type; (3) contrary to our predictions, low‐disturbance ecosystems (native forest) did not appear to host a significant source population of spiders in productive landscapes (kiwifruit); however, (4) edge effects between land‐uses did create different spider community assemblages along the 50 m transect into the land‐use, particularly for different spider life‐stages. More immature spiders were observed at the edges of the productive land crops and this significantly dropped off towards the 50 m centre of the kiwifruit crops, whilst adult numbers were relatively stable throughout the transect. This finding suggests there is potential for adjacent land‐use to host a source population of spiders in productive systems, and more studies are needed to ascertain whether factors such as increased landscape connectivity, reduced synthetic management inputs, or within‐crop non‐crop plantings can enhance retention of spiders.

Given that kiwifruit is a significant economic crop for Aotearoa New Zealand, that pressure is mounting to move away from synthetic insecticide inputs in favour of IPM practises, and that spiders present significant pest control potential, we encourage further studies in this area.

## Author Contributions

**Nicola J. Sullivan:** conceptualization (equal), data curation (equal), funding acquisition (lead), investigation (lead), methodology (lead), project administration (lead), writing – original draft (lead), writing – review and editing (lead). **Lloyd D. Stringer:** conceptualization (equal), funding acquisition (supporting), investigation (supporting), project administration (supporting), supervision (equal), writing – review and editing (supporting). **Ruth C. Butler:** data curation (supporting), formal analysis (lead), methodology (supporting), writing – review and editing (supporting). **Amanda Black:** conceptualization (supporting), funding acquisition (supporting), project administration (supporting), supervision (supporting), writing – review and editing (supporting). **Nick Waipara:** funding acquisition (supporting), resources (supporting), supervision (supporting), writing – review and editing (supporting). **Cor J. Vink:** conceptualization (equal), funding acquisition (supporting), investigation (supporting), methodology (supporting), project administration (supporting), supervision (equal), writing – review and editing (supporting).

## Funding

This work was supported by Bio‐Protection Research Centre. Food Transitions 2050, PhD scholarship. Ministry of Business, Innovation and Employment, Strategic Science Investment Fund.

## Ethics Statement

Kiwifruit Vine Health (KVH) approval was given for this study on 14th December 2022 with conditions in place to ensure disinfection processes were carried out to prevent the transmission of *Pseudomonas syringae* pv. *actinidiae* (Psa, a bacterium that can result in the death of susceptible kiwifruit varieties).

## Conflicts of Interest

The authors declare no conflicts of interest.

## Supporting information


**Table S1:** Functional guilds of spider families according to Cardoso et al. (2011).
**Table S2:** A list of species collected from kiwifruit orchards, native forest, and maize crops in Te Kaha, Aotearoa New Zealand.
**Table S3:** Total adult spiders caught by guild, family and species.
**Table S4:** Number of each spider family found at each assessment along with the totals for all assessments and that for assessments two to four. Data are ordered by overall total. Groups are S small (Overall < 30 individuals), M mid (30 to 250 individuals), L large (> 250 individuals).
**Table S5:** Total spiders found for each of eight functional guilds according to Cardoso et al. (2011).
**Figure S1:** Mean total numbers of spiders caught for the entire trial, percentage that were adults or males, and two diversity statistics (Shannon–Wiener Index [Shannon]; Smith & Wilson Evenness [Evar]) for Aotearoa New Zealand kiwifruit orchards, as obtained from the fitted model, for maize and native forest, by sampling method and land‐use type. Error bars are 95% confidence intervals. The upper limit for a mean of 0 is difficult to obtain so is not shown.

## Data Availability

The data that support the findings of this study are openly available in Zenodo at http://doi.org/10.5281/zenodo.17460985.
