# Supplementary material for: Spider (Araneae) Abundance and Diversity Across Land‐Use Gradients
Source: Ecol Evol. 2026 Sep 16;16(9):e74261. doi: 10.1002/ece3.74261 (PMC13583177; doi:10.1002/ece3.74261)
Supplement: Supplementary file 1 [file ECE3-16-e74261-s001.zip › ece374261-sup-0001-Supinfo01.docx]

Supplementary material

Supplementary Table 1. Functional guilds of spider families according to Cardoso et al. (2011).

| **Family** | **Functional Guild (Cardoso et al. 2011)** |
| --- | --- |
| Anapidae | Orb weaver |
| Araneidae | Orb weaver |
| Corinnidae | Ground hunter |
| Clubionidae | Other hunter |
| Cyatholipidae | Sheet web weaver |
| Desidae | Ground hunter |
| Dysderidae | Specialist |
| Gnaphosidae | Ground hunter |
| Hahniidae | Sheet web weaver |
| Lamponidae | Specialist |
| Linyphiidae | Sheet web weaver |
| Lycosidae | Ground hunter |
| Mimetidae | Specialist |
| Pycnothelidae | Sensing web weaver |
| Oxyopidae | Other hunter |
| Pisauridae | Sheet web weaver |
| Physoglenidae | Orb weaver |
| Salticidae | Other hunter |
| Stiphidiidae | Sheet web weaver |
| Theridiidae | Space web weaver |
| Theridiosomatidae | Orb weaver |
| Tetragnathidae | Orb weaver |
| Thomisidae | Ambush hunter |
| Toxopidae | Sheet web weaver |
| Zoropsidae | Sheet web weaver |

Supplementary Table 2. A list of species collected from kiwifruit orchards, native forest, and maize crops in Te Kaha, Aotearoa New Zealand.

| Family | Species | Adults | Family | Species | Adults |
| --- | --- | --- | --- | --- | --- |
| Anapidae | *Paranapsis insula* | 3 | Linyphiidae | *Erigone prominens* | 100 |
| Araneidae | *Celania* sp. | 3 | Linyphiidae | *Tenuiphantes tenuis* | 128 |
| Araneidae | *Celania atkinsoni* | 2 | Desidae | *Badumna longuina* | 14 |
| Desidae | Desidae sp. | 2 | Salticidae | *Maratus griseus* | 14 |
| Gnaphosidae | *Intruda signata* | 2 | Theridiidae | *Cryptachaea blattea* | 14 |
| Gnaphosidae | *Scotophaeus pretiosus* | 2 | Theridiidae | Theridiidae spp. | 15 |
| Linyphiidae | *Laetesia peramoena* | 2 | Theridiidae | Theridiidae sp. pale, black spots | 15 |
| Pisauridae | *Dolomedes minor* | 2 | Theridiidae | *Steatoda capensis* | 31 |
| Salticidae | *Trite* spp. new species | 2 | Theridiidae | *Cryptachaea veruculata* | 37 |
| Theridiidae | *Argyrodes antipodiana* | 2 | Lycosidae | *Anoteropsis hilaris* | 51 |
| Thomisidae | *Diaea* sp. | 2 | Tetragnathidae | *Leucauge dromedaria* | 59 |
| Araneidae | *Salsa fuliginata* | 1 | Salticidae | *Trite auricoma* | 3 |
| Araneidae | *Poecilopachys australasia* | 1 | Stiphidiidae | *Stiphidion facetum* | 3 |
| Araneidae | *Zealaranea crassa* | 1 | Theridiidae | *Phoroncidia* sp. | 3 |
| Cyatholipidae | *Tekelloides flavonotatus* | 1 | Theridiidae | *Moneta conifera* | 3 |
| Lamponidae | *Lampona murina* | 1 | Thomisidae | Thomisidae Sp. | 3 |
| Linyphiidae | *Erigone wiltoni* | 1 | Gnaphosidae | *Anzacia gemmea* | 4 |
| Linyphiidae | *Laetesia trispathulata* | 1 | Salticidae | *Helpis minitabunda* | 4 |
| Lycosidae | *Venatrix goyderi* | 1 | Physoglenidae | *Pahora* sp. | 5 |
| Mimetidae | *Australomimetus* sp. | 1 | Tetragnathidae | *Tetragnatha* sp. | 5 |
| Salticidae | *Holoplatys apressus* | 1 | Theridiidae | *Steatoda grossa* | 7 |
| Salticidae | *Trite planiceps* | 1 | Araneidae | *Socca pustulosa* | 8 |
| Theridiidae | *Steatoda lepida* | 1 | Thomisidae | *Sidymella longipes* | 8 |
| Theridiidae | *Moneta conifera* | 1 | Desidae | *Badumna insignis* | 9 |
| Theridiidae | *Phycosoma oecobioides* | 1 | Stiphidiidae | *Cambridgea quadromaculata* | 10 |
| Theridiidae | *Theridion zantholabio* | 1 | Araneidae | Araneidae sp. new species | 11 |
| Theridiosomatidae | *Baalzebub* sp. | 1 | Pycnothelidae | *Stanwellia bipectinata* | 12 |
| Toxopidae | Toxopidae sp. | 1 | Zoropsidae | *Uliodon* sp. | 13 |
| Oxyopidae | *Oxyopes gracilipes* | 0 | Araneidae | *Novakiella trituberculosa* | 3 |
| Cycloctenidae | Cycloctenidae sp. | 0 | Corinnidae | *Nyssus coloripes* | 3 |
| Idiopidae | Idiopidae sp. | 0 | Clubionidae | *Clubiona contrita* | 3 |
| Micropholcommatidae | Micropholcommatidae sp. | 0 | Hahniidae | *Rinawa* sp. | 3 |

Supplementary Table 3. Total adult spiders caught by guild, family and species.

| Guild | Family | Species | Total Spiders |
| --- | --- | --- | --- |
| Ambush hunter | Thomisidae | *Diaea* sp. | 2 |
|  |  | *Sidymella longipes* | 8 |
|  |  | Thomisidae sp. | 3 |
| Ground hunter | Corinnidae | *Nyssus coloripes* | 3 |
|  | Cycloctenidae | Cycloctenidae sp. | 0 |
|  | Desidae | *Badumna insignis* | 9 |
|  |  | *Badumna longuina* | 14 |
|  |  | Desidae sp. | 2 |
|  | Gnaphosidae | *Anzacia gemmea* | 4 |
|  |  | *Intruda signata* | 2 |
|  |  | *Scotophaeus pretiosus* | 2 |
|  |  | Gnaphosidae sp. | 0 |
|  | Lycosidae | *Anoteropsis hilaris* | 51 |
|  |  | *Venatrix goyderi* | 1 |
| Orb weaver | Anapidae | *Paranapsis insula* | 3 |
|  |  | *Celania atkinsoni* | 2 |
|  |  | *Celania* sp. | 3 |
|  |  | *Novakiella trituberculosa* | 3 |
|  |  | *Poecilopachys australasia* | 1 |
|  |  | *Salsa fuliginata* | 1 |
|  |  | *Socca pustulosa* | 8 |
|  |  | Araneidae sp. new species | 11 |
|  |  | *Zealaranea crassa* | 1 |
|  | Physoglenidae | *Pahora* sp. | 5 |
|  | Tetragnathidae | *Leucauge dromedaria* | 59 |
|  |  | *Tetragnatha* sp. | 5 |
|  | Theridiosomatidae | *Baalzebub* sp. | 1 |
| Other hunter | Clubionidae | *Clubiona contrita* | 3 |
|  | Oxyopidae | *Oxyopes gracilipes* | 0 |
|  | Salticidae | *Helpis minitabunda* | 4 |
|  |  | *Holoplatys apressus* | 1 |
|  |  | *Maratus griseus* | 14 |
|  |  | *Trite auricoma* | 3 |
|  |  | *Trite* spp. new species | 2 |
|  |  | *Trite planiceps* | 1 |
| Sensing web weaver | Idiopidae | Idiopidae sp. | 0 |
|  | Pycnothelidae | *Stanwellia bipectinata* | 12 |
| Sheet web weaver | Cyatholipidae | *Tekelloides flavonotatus* | 1 |
|  | Hahniidae | *Rinawa* sp. | 3 |
|  | Linyphiidae | *Erigone prominens* | 101 |
|  |  | *Erigone wiltoni* | 1 |
|  |  | *Laetesia peramoena* | 2 |
|  |  | *Laetesia trispathulata* | 1 |
|  |  | *Ostearius melanopygius* | 0 |
|  |  | *Tenuiphantes tenuis* | 130 |
|  | Pisauridae | *Dolomedes minor* | 2 |
|  |  | *Cambridgea quadromaculata* | 10 |
|  |  | *Stiphidion facetum* | 3 |
|  |  | Stiphidiidae sp. | 0 |
|  | Toxopidae | Toxopidae sp. | 1 |
|  | Zoropsidae | *Uliodon* sp. | 13 |
| Space web weaver | Micropholcommatidae | Micropholcommatidae sp. | 0 |
|  | Theridiidae | *Argyrodes antipodiana* | 2 |
|  |  | *Cryptachaea blattea* | 14 |
|  |  | *Cryptachaea veruculata* | 37 |
|  |  | *Moneta conifera* | 1 |
|  |  | *Moneta conifera* | 3 |
|  |  | Theridiidae sp. pale, black spots | 16 |
|  |  | *Phoroncidia* sp. | 3 |
|  |  | *Phycosoma oecobioides* | 1 |
|  |  | Theridiidae sp. | 15 |
|  |  | *Steatoda capensis* | 31 |
|  |  | *Steatoda grossa* | 7 |
|  |  | *Steatoda lepida* | 1 |
|  |  | *Theridion zantholabio* | 1 |
| Specialist | Lamponidae | *Lampona murina* | 1 |
|  | Mimetidae | *Australomimetus* sp. | 1 |

Supplementary Table 4. Number of each spider family found at each assessment along with the totals for all assessments and that for assessments two to four. Data are ordered by overall total. Groups are S small (Overall<30 individuals), M mid (30 to 250 individuals), L large (>250 individuals).

|  | 23 |  | 24 |  | Totals |  |  |
| --- | --- | --- | --- | --- | --- | --- | --- |
| Family | Jan | Apr | Jan | Apr | Apr23 – Apr24 | Overall | Group |
| Lamponidae | 0 | 1 | 0 | 0 | 1 | 1 | S |
| Micropholcommatidae | 0 | 0 | 1 | 0 | 1 | 1 | S |
| Mimetidae | 0 | 1 | 0 | 0 | 1 | 1 | S |
| Toxopidae | 0 | 1 | 0 | 0 | 1 | 1 | S |
| Idiopidae | 0 | 0 | 0 | 2 | 2 | 2 | S |
| Corinnidae | 2 | 1 | 0 | 0 | 1 | 3 | S |
| Hahniidae | 0 | 3 | 0 | 0 | 3 | 3 | S |
| Theridiosomatidae | 0 | 2 | 0 | 1 | 3 | 3 | S |
| Cyatholipidae | 0 | 0 | 0 | 5 | 5 | 5 | S |
| Physoglenidae | 0 | 5 | 1 | 2 | 8 | 8 | S |
| Anapidae | 3 | 6 | 0 | 0 | 6 | 9 | S |
| Pisauridae | 4 | 8 | 3 | 1 | 12 | 16 | S |
| Oxyopidae | 11 | 1 | 5 | 0 | 6 | 17 | S |
| Zoropsidae | 1 | 9 | 0 | 9 | 18 | 19 | S |
| Clubionidae | 1 | 8 | 4 | 7 | 19 | 20 | S |
| Pycnothelidae | 7 | 9 | 2 | 2 | 13 | 20 | S |
| Gnaphosidae | 7 | 6 | 5 | 4 | 15 | 22 | S |
| Cycloctenidae | 5 | 5 | 12 | 11 | 28 | 33 | M |
| Thomisidae | 7 | 14 | 18 | 11 | 43 | 50 | M |
| Salticidae | 8 | 36 | 13 | 28 | 77 | 85 | M |
| Lycosidae | 48 | 12 | 29 | 13 | 54 | 102 | M |
| Stiphidiidae | 23 | 40 | 20 | 39 | 99 | 122 | M |
| Desidae | 49 | 38 | 80 | 35 | 153 | 202 | M |
| Linyphiidae | 125 | 39 | 84 | 49 | 172 | 297 | L |
| Tetragnathidae | 27 | 134 | 101 | 153 | 388 | 415 | L |
| Theridiidae | 45 | 164 | 96 | 138 | 398 | 443 | L |
| Araneidae | 120 | 189 | 188 | 313 | 690 | 810 | L |

Supplementary Table 5. Total spiders found for each of eight functional guilds according to Cardoso et al. (2011).

| Guild | Total found | % |
| --- | --- | --- |
| Orb weaver | 1245 | 45.9 |
| Sheet web weaver | 463 | 17.1 |
| Space web weaver | 444 | 16.4 |
| Ground hunter | 362 | 13.4 |
| Other hunter | 122 | 4.5 |
| Ambush hunter | 50 | 1.8 |
| Sensing web weaver | 22 | 0.8 |
| Specialist | 2 | 0.1 |
| Total | 2710 |  |

**Supplementary Figure 1**. Mean total numbers of spiders caught for the entire trial, percentage that were adults or males, and two diversity statistics (Shannon-Wiener Index [Shannon]; Smith & Wilson Evenness [Evar]) for Aotearoa New Zealand kiwifruit orchards, as obtained from the fitted model, for maize and native forest, by sampling method and land-use type. Error bars are 95% confidence intervals. The upper limit for a mean of 0 is difficult to obtain so is not shown.
